# Channelling the Force to Reprogram the Matrix: Mechanosensitive Ion Channels in Cardiac Fibroblasts

**DOI:** 10.3390/cells10050990

**Published:** 2021-04-23

**Authors:** Leander Stewart, Neil A. Turner

**Affiliations:** Discovery and Translational Science Department, Leeds Institute of Cardiovascular and Metabolic Medicine, School of Medicine, University of Leeds, Leeds LS2 9JT, UK; l.stewart@leeds.ac.uk

**Keywords:** ion channels, mechanical signalling, mechanosensation, cardiac fibroblasts, cardiac remodelling, extracellular matrix, fibrosis, TRP channels, Piezo1, potassium channels

## Abstract

Cardiac fibroblasts (CF) play a pivotal role in preserving myocardial function and integrity of the heart tissue after injury, but also contribute to future susceptibility to heart failure. CF sense changes to the cardiac environment through chemical and mechanical cues that trigger changes in cellular function. In recent years, mechanosensitive ion channels have been implicated as key modulators of a range of CF functions that are important to fibrotic cardiac remodelling, including cell proliferation, myofibroblast differentiation, extracellular matrix turnover and paracrine signalling. To date, seven mechanosensitive ion channels are known to be functional in CF: the cation non-selective channels TRPC6, TRPM7, TRPV1, TRPV4 and Piezo1, and the potassium-selective channels TREK-1 and K_ATP_. This review will outline current knowledge of these mechanosensitive ion channels in CF, discuss evidence of the mechanosensitivity of each channel, and detail the role that each channel plays in cardiac remodelling. By better understanding the role of mechanosensitive ion channels in CF, it is hoped that therapies may be developed for reducing pathological cardiac remodelling.

## 1. Introduction

### 1.1. The Heart and Its Cellular Constituents

Cardiac pathologies that arise from cardiac injury or dysfunction vastly increase the probability of heart failure (HF) and are a leading cause of morbidity and mortality worldwide [1,2]. Many of those affected have fibrotic heart disease, which involves over-production of extracellular matrix (ECM) proteins, particularly type I and II fibrillar collagens, resulting in stiffening of the heart, with resultant impairment of cardiac contraction and relaxation, and interference of electrical signalling. Advancements in our understanding of the complex cellular composition and communication within the heart, and functionality and malleability of the phenotype of each cardiac cell subpopulation, have highlighted the importance of cardiac cellular diversity in the maintenance of normal cardiac function and in the response to pathology.

Cardiomyocytes (CM) are considered to be the primary regulators of cardiac function and this cell type makes up between one-third and a half of the total cell population [3,4,5,6]. CM are excitable cells which form the cardiac muscle and are responsible for the contractile forces of the heart. While much cardiac research has naturally been focused on CM, in recent decades, the importance of non-myocyte cells within the heart has gained traction. One of the most prevalent non-myocyte cell types in the heart is the cardiac fibroblast (CF), accounting for an estimated 10–30% of total cells in the rodent and human heart [3,4,5,6], although precise quantification is confounded by the heterogenous nature of this cell population. The proportion of fibroblasts increases substantially in the pathologically remodelling heart [6]. CF had once been considered a more passive cell type, functioning only to regulate remodelling of the ECM [7]. However, in more recent years, an upsurge of interest in how fibroblasts influence the progression of cardiac pathologies has exemplified the importance of CF in the response of the myocardium to pathological assault.

### 1.2. Cardiac Fibroblasts

Within normally functioning healthy hearts, CF are mostly quiescent and well embedded within the structurally stable ECM [8]. However, during cardiac injury or pathological progression, the phenotypical characteristics of CF become malleable, as they respond to stress cues and adapt to the changing environment. Under pathological conditions fibroblasts transdifferentiate into myofibroblasts (MF), developing more proliferative and migratory capabilities, contractile qualities, and enhanced secretion of ECM proteins, growth factors and cytokines in order to regulate the local cellular environment via paracrine signalling [7]. A key characteristic of the MF phenotype is expression of alpha-smooth muscle actin (α-SMA), a cytoskeletal protein which forms highly contractile microfilaments; expression of which enables the contractile nature of MF [9]. The CF-to-MF response to injury represents a protective and reparative mechanism which acts to preserve cardiac function; and in the case of pressure-induced ventricular overload (pressure overload; PO), prevents ventricular wall rupture through production of interstitial fibrosis and promotion of CM hypertrophy [10,11].

Following myocardial infarction (MI), MF proliferate and migrate to the site of ischemic injury and regulate scar formation by reparative and replacement fibrosis; through production of ECM proteins, particularly collagens type I and III and fibronectin [9], and crosslinking of collagen fibres to generate stiffer matrices, which are less compliant to applied forces and more resistant to deformation [8]. The fibrotic scar replaces damaged and/or dying CM, maintaining the cardiac tissue integrity during the pathological assault. MF further fine-tune ECM turnover through highly regulated secretion and activation of ECM-degrading proteases such as matrix metalloproteinases (MMPs) and their endogenous inhibitors, tissue inhibitors of metalloproteinases (TIMPs) [12].

### 1.3. Mechanical Activation of Cardiac Fibroblasts

**Figure 1:**
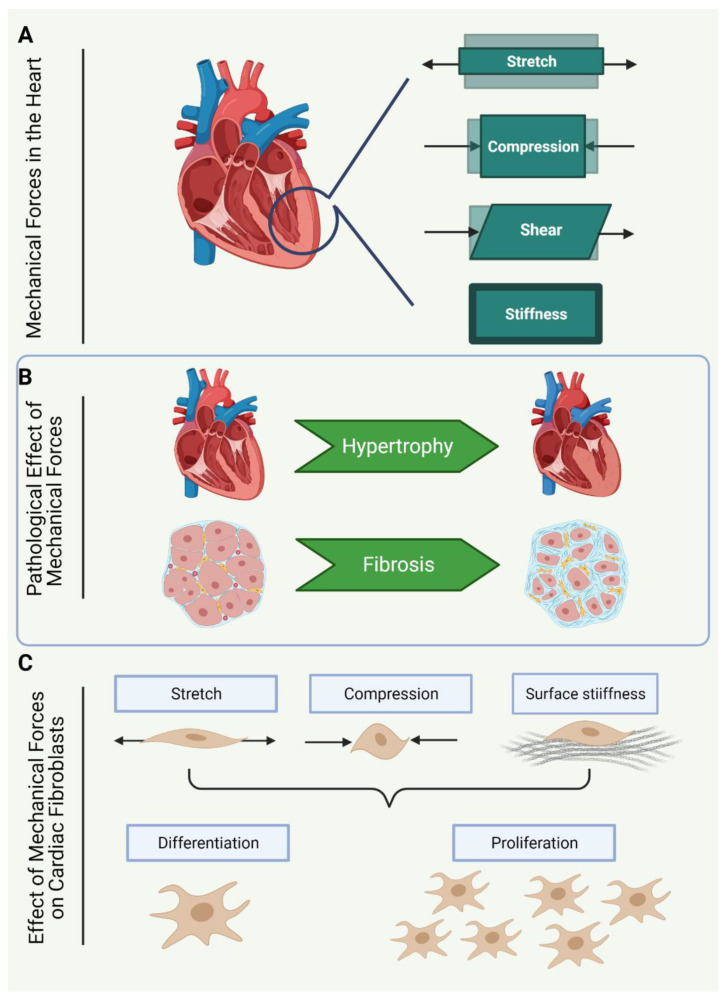
The pathophysiological effect of mechanical forces on the heart. (**A**) The heart is subject to mechanical forces such as stretch and compression as a consequence of the rhythmic beating of the heart, shear stress due to blood flow, and tension due to the stiffening of tissues under certain physiological and pathophysiological conditions. (**B**) Mechanical forces which occur during pathophysiological conditions prompt cardiac fibrosis and cardiomyocyte hypertrophy, resulting in stiffening and thickening of the muscular walls of the heart. (**C**) Cardiac fibroblasts are thought to be sensitive to the changes in mechanical forces of the heart. In culture, cardiac fibroblasts are sensitive to the stiffness of the culture surface and tensile forces, such as stretch and compression. In response to tensile forces and surface stiffness, cardiac fibroblasts transdifferentiate into myofibroblasts and proliferate more rapidly.

The contractile nature of the heart exerts multiple forms of mechanical forces on the cardiac cell population with every heartbeat. The routine contraction and filling of the heart chambers induce three-dimensional and non-uniform deformation of the heart tissue, causing mechanical strain and tensile forces as the tissue is stretched and compressed (Figure 1A). Specific regions of the heart are also subjected to shearing forces due to friction caused by filling and ejection of blood volume [8].

Under normal physiological conditions, the structural stability of the ECM is thought to offer the CF a certain amount of protection from any substantial changes to the forces of the beating heart [13]. However, during the progression of cardiac pathologies such as PO and MI, the mechanical strain imposed on the ECM, CF, and CM prompts activation of the ECM [8,13], as well as the CF population [8,13]. The interplay between the ECM and CF in response to altered mechanical stimuli appears important for progression of the fibrotic response to pathology. CF stimulate the initial breakdown of the ECM, resulting in activation and release of profibrotic signalling molecules, such as transforming growth factor β (TGF-β), and some extracellular damage-associated molecular patterns (DAMPs), such as SLRPs, syndecans, glypicans and hyaluronic synthases [14,15,16,17,18]. While current understanding of the initiating events of cardiac fibrosis suggests CF activation prompts release of profibrotic signalling molecules from the ECM reservoir, Herum and colleagues propose that some extracellular DAMPs are activated in response to a high level of mechanical strain on the cardiac ECM, and subsequently drive activation of CF [8]. The loss of ECM structural protection potentially increases CF sensitivity to mechanical forces, further promoting the MF phenotype through re-expression of foetal gene pathways, and driving modulation of the cytoskeleton and surrounding ECM [19].

Once differentiated into the MF phenotype, MF initiate further remodelling of the ECM, altering the composition and stiffness of the matrix. As part of such remodelling, an increase in fibrillary collagens (collagen type I and III) and crosslinking of collagen fibres generate stiffer matrices that are less compliant to applied forces and more resistant to deformation [20,21,22]. CF tethered to the ECM sense changes to the stiffness of the ECM and adherence of fibroblasts to stiffer substrates further supports the profibrotic MF phenotype [23].

### 1.4. Mechanical Forces Sensed by Cardiac Fibroblasts

In order to understand the mechanosensitivity of CF, it is important to recognise the types of mechanical stimuli that CF respond to (Figure 1C). It is worth noting, however, that our current understanding of CF responses to mechanical stimuli are limited by experimental parameters, and thus much of what is known in this regard is based on in vitro models. In vitro, CF can respond to multiple types of mechanical forces, including mechanical strain, which describes the deformation of the plasma membrane through stretch [24], cyclic stretch [25] or compression [26,27]. Additionally, CF sense mechanical stimuli via ECM contact with cell surface proteins, such as focal adhesion protein complexes, which tether to the cytoskeleton [28]. CF can also sense cell traction forces from neighbouring cells, via adhesion receptors, and through tension sensed by the phospholipid bilayer of the plasma membrane [19].

Changes to the composition of the ECM alter how CF interact with the ECM, through changes to the number of contact points, changes to integrin contacts with the ECM, and the complex formation of focal adhesions [28], and may also drive changes to the shape of the nucleus [29]. Alterations in the shape of the nucleus in response to CF adherence to a stiff substrate can directly alter gene expression patterns [30,31].

Integrins are a family of heterodimeric proteins that act as primary regulators of contact between cells and the ECM. Upon integrin binding to extracellular ligands, integrins and integrin adhesome proteins cluster at sites of cell–ECM contacts, forming focal adhesion complexes [32]. Integrins and their focal adhesion complexes have been well described as important sensors of mechanical stimulation in CF, with extensive research outlining the importance of these proteins in driving CF-to-MF transdifferentiation [33]. In non-CF cell types, integrins have also been shown to interact with stretch-sensitive cation channels [34,35], adding further dynamics to their ability to respond to mechanical stimuli. Note that reference [34] is a pre-print and has not yet been peer-reviewed. Integrins may further regulate the activation of CF via crosstalk with other signalling molecules, such as TGF-β, membrane-bound receptors and tyrosine kinase receptors [33].

Deformation of the cell membrane also provides cues for cardiac fibrosis through promoting cellular ion influx, in particularly influx of Ca^2+^ ions [36]. The pivotal role of such mechanosensitive cation channels in altering CF function forms the topic of this review.

### 1.5. Cardiac Fibroblasts in Culture

Studies which strive to understand how biomechanical forces influence the CF phenotype and functionality have been complicated by the sensitivity of CF to many aspects of the artificial in vitro environment [37,38,39,40,41,42,43,44,45,46,47,48,49]. CF are highly sensitive to tissue stiffness and elasticity and spontaneously transdifferentiate into proto-myofibroblasts when grown in culture [23]. The stiffness of a substrate can be described using Young’s modulus (measured in Pascals, Pa), which quantifies the resistance of a material to deformation when under stress [50]. Under normal physiological conditions, tissue of the heart has a Young’s modulus of ~10 kPa, but under fibrotic pathophysiological conditions this increases to 20–100 kPa [51]. Conventional polystyrene tissue culture plates have a Young’s modulus of around 1 million kPa, representing a vastly elevated substrate stiffness compared to that encountered in the heart. To maintain the fibroblast phenotype in tissue culture, CF can be grown on softer substrates, such as hydrogels [8].

Many studies have attempted to recapitulate the cardiac environment in vitro through use of tissue culture models, which combine multidimensional characteristics of the heart; such as specific tissue culture matrix composition and stiffness, cyclic stretch, and biochemical modulators of CF [25,52,53,54,55]. In vitro analysis of cells cultured in systems which generate cyclic stretch has revealed that CF respond differently according to the strength of the stretch, the duration, and other modifications to the cellular environment (i.e., substrate/matrix composition and stiffness, and oxygenation) [25,52,53,54,55]. Given these observations, it is important to consider the nature of the cell culture environment when evaluating mechanosignalling research in CF.

### 1.6. Mechanosensitive Cation Channels in Cardiac Fibroblasts

CF express multiple cation channels that enable fluxes of cations (predominantly Ca^2+^, Mg^2+^, Na^+^ and K^+^) across the cell membrane. In vitro analysis of rat CF has demonstrated that these cells are not electrically excitable [56], but instead experience mechanically-induced membrane potential oscillations [26]. In situ CF cation conductance is sensitive to the heartbeat, with atrial relaxation prompting hyperpolarization of CF membrane potential, and atrial contraction driving depolarization [26,57,58,59,60,61]. Mechanosensitive ion channels are capable of responding to mechanical stress imposed on the cell; for example, alterations in membrane curvature and thickness, and in-plane membrane tension [62]. Such forces can occur as a consequence of shear fluid force, osmotic swelling of the cell, surface tension (through matrix–protein interaction), and compression of the cell [62]. Activation of such channels enables ion flux and subsequent changes to cellular activity, eliciting the cells ability to respond and adapt to their environment through prompting changes in gene expression and cellular remodelling [62].

There are numerous cation channels expressed in the heart, but only a small number are considered to be mechanically gated and functional within CF, indicating a potential role for ion conductance in response to mechanical stimuli. These channels include cation non-selective channels (TRPC6, TRPM7, TRPV1, TRPV4, and Piezo1), as well as potassium-selective channels (TREK-1 and KATP). However, it should be noted that the concept of TRP channels acting as primary sensors of mechano-stimuli is the subject of much debate [63]. While ample evidence exists supporting their function in cellular responses to mechano-stimulation [64], more recent studies suggest TRP channels may not function as direct sensors of mechano-stimuli [65], but rather act downstream of other primary sensors of mechanical stimulation [66,67]. While these seven channels have been selected due to evidence supporting their direct mechanosensitivity, it should also be noted that large-conductance, Ca^2+^ and voltage-activated potassium (BK_Ca_) channels have also been described to be important in CF biology [68,69] and may also respond to membrane stretch. Expression of stretch-activated BK_Ca_ channels has been detected at low levels in atrial fibroblasts, however, patch clamp measurements of the stretch-induced currents in these cells were found to be largely dependent on Piezo1 activity [69]. For other isoforms of BK_Ca_ channels, their response to stretch has been demonstrated to be indirect and instead driven by stretch-evoked increase in intracellular Ca^2+^ levels [62,69,70]; a concept further supported by selective chemical inhibition of BK_Ca_ channels which failed to modify stretch evoked currents in vagal mechanically-sensitive afferents in guinea-pig oesophagus [71]. Thus, BK_Ca_ will not be described in detail within this review.

These five non-selective ion channels and two potassium-selective channels will form the basis of this review and will be discussed in subsequent sections initially in general terms, with emphasis on evidence outlining the mechanosensitivity of each channel (Table 1), before exploring their function in fibrotic cardiac remodelling and ultimately their known role in CF.

## 2. Transient Receptor Potential (TRP) Channels

TRP channels are widely expressed integral membrane proteins that are responsive to many different types of stimuli such as light, heat, mechanical stress, and a number of different chemical ligands [83]. There are 28 known TRP-related genes in mammals, which can be grouped into six families: TRPA (ankyrin), TRPC (canonical), TRPM (melastatin), TRPML (mucolipin), TRPP (polycystin or polycystic kidney disease) and TRPV (vanilloid). The specific cations that a channel permeates differs depending on the specific type of TRP channel [83]. The majority of the mammalian expressed TRP channels regulate Ca^2+^ influx, with their activity influencing microdomain signalling and endoplasmic reticulum Ca^2+^ store reloading; with the exception of TRPM4 and TPRM5 which are activated by Ca^2+^, but are not Ca^2+^-permeable [84]. The TRP family of ion channels has been suggested to function in transduction of mechanosignalling in flies, worms, and mammals [85]. As previously mentioned, more recent studies have called into question whether the TRP family of cation channels are truly mechanosensitive. For example, single-channel conductance measurements of the mammalian TRP ion channels, using patch clamping applied pipette pressure, revealed that none of the TRP channels are stretch sensitive within a heterologous expression system [65]. An up-to-date and detailed overview of our current understanding of TRP channels is available in other recent review articles [83,86].

### 2.1. Canonical Family of Transient Receptor Potential (TRPC) Channels

The TRPC family comprises seven members that are widely expressed in most types of cardiac cells, although it should be noted that TRPC2 is expressed in mice but not humans [87]. TRPC channels appear to be important during cardiac dysfunction, with almost all TRPC channels (excluding TRPC2 and TRPC7) upregulated during HF in humans [88,89]. All TRPC channels are regulated by phospholipase C (PLC), and are grouped into two subfamilies based on structural and functional similarities: TRPC3/6/7, which co-ordinate in response to the secondary lipid messenger, diacylglycerol (DAG) [90,91]; and TRPC1/4/5, which are insensitive to DAG [92]. TRPC3, 6 and 7 are involved in receptor-mediated Ca^2+^ entry, while TRPC1, 4 and 5 regulate store-operated Ca^2+^ entry [93,94,95].

The TRPC3/6/7 channels form hetero-tetramer channels [90,91] that are permeable to Na^+^, and thus are important for signalling during depolarization. The channels also form pores which are moderately permeable to Ca^2+^ under normal physiological conditions [96], and more highly permeable to Ca^2+^ under pathophysiological conditions, and are important mediators during fibrosis [96]. In addition to DAG, TRPC3/6/7 are activated by a number of other mechanisms, including binding of specific lipid molecules and PLC through Gα_q/11_ protein-coupled receptors, interaction with receptor tyrosine kinases, and by the vasoconstrictors noradrenaline and Arg8-vasopressin. TRPC3/6/7 are also important in responding to mechanical stimuli such as stretch, flow and osmotic pressure [97].

**Figure 2:**
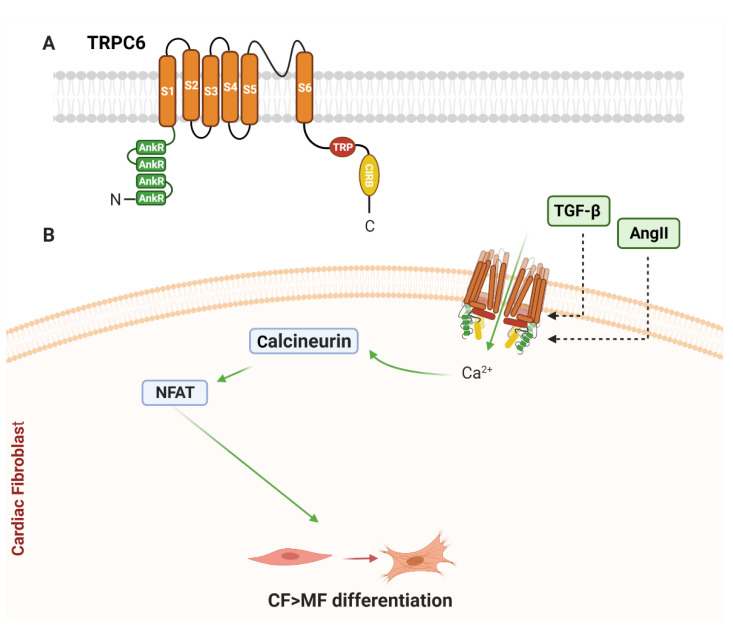
Schematic diagram of TRPC6 structure and signalling in cardiac fibroblasts. (**A**) Basic structure of alpha subunit of TRPC6. Each TRPC6 alpha subunit is comprised of six transmembrane-spanning domains (S1–S6), with a pore loop between S5 and S6, ankyrin repeats (AnkR) at the amino terminus, and a TRP-box (TRP) and calmodulin- and IP3 receptor-binding (CIRB) domain at the carboxyl termini. (**B**) Known TRPC6 homotetrameric ion channel signalling in cardiac fibroblasts. Ang II and TGF-β can upregulate cardiac fibroblast TRPC6 gene expression resulting in increased Ca^2+^ influx. TRPC6-mediated Ca^2+^ entry is coupled to cardiac myofibroblast differentiation via activation of the calcineurin–NFAT signalling pathway. See main text for details.

While all TRPC channels are expressed in the heart, only TRPC1 and TRPC6 have been reported to be mechanosensitive. As TRPC1 expression is not detected in CF [98], the following section will focus on TRPC6 (Figure 2A). For a more detailed review of all TRPC channels, please refer to [99].

Transgenic mouse models have highlighted the importance of TRPC6 for sensations linked to neuronal mechanotransduction; such as touch and hearing [74]. In vitro evidence has further supported the notion of TRPC6 mechanosensitivity, with over-expression of TRPC6 in a heterologous system activating in response to membrane thinning and stretch [72] (Table 1). However, such findings have been subsequently contradicted [65,66], calling into question the true mechanosensitivity of these channels. Nikolaev et al. reported that TRPC6 does not respond directly to stretching of the membrane itself, but rather responds to tension generated by cytoplasmic tethers, thus acting as a downstream mediator in response to mechanical stimulation [65]. Further studies have suggested that TRPC6 becomes mechanosensitive once active, and that the channel may require activation of the PLA_2_/ω-hydroxylase metabolite 20-HETE pathway to participate in the response to mechano-stimuli [100]. Taken together, it can be considered that TRPC channels do not act as primary sensors of mechano-stimuli, but are still important in the cellular response to such stimuli.

#### 2.1.1. TRPC6 and Cardiac Remodelling

The function of the cardiac TRPC6 channel is complex, and while TRPC6 function may protect against cardiac injury, it also correlated with an increase in inflammation, fibrosis, and poor prognosis following cardiac injury and PO [101,102,103,104].

TRPC6 promotes wound healing and prevents rupture of the ventricular wall during PO [101]. Global TRPC6 knockout (KO) mice had reduced LV fractional shortening and increased LV end diastolic dimensions following MI compared to WT littermates, resulting in reduced cardiac function. Further to this, global TRPC6 KO mice had reduced survival following right ventricle PO due to an increased risk of ventricle wall rupture [101]. However, TRPC6 global KO mice that survived LV PO had smaller scar sizes and reduced fibrosis when compared to their WT littermates [101]. Conversely, Oda et al. demonstrated that global deletion of TRPC6 in mice had no impact on PO following transverse aortic constriction (TAC) surgery, despite loss of TRPC6 leading to a reduction in interstitial fibrosis [90]. TRPC6 KO had little effect on production of ROS and expression of fibrotic markers at the mRNA level, but did lead to an increase in inflammatory cytokines [90].

Chemical inhibition of TRPC6 by BI 749327 in mice that have undergone TAC also led to a reduction in cardiac fibrosis, but did not impact on cardiac hypertrophy [105]. Oda et al. hypothesised that TRPC6 depletion in mouse CF may be beneficial in reducing PO-induced fibrosis, but inhibition of TRPC6 expression in CM may exasperate cardiac dysfunction after PO [90]. The differential roles of TRPC6 in different cardiac cells may therefore complicate pharmacological targeting of this channel in the heart.

Over-expression of TRPC6 in mice, under the α-MHC promoter, prompts an increase in TRPC6 expression that signals through a calcineurin–NFAT signalling module and increases sensitivity to stress, pathological cardiac growth, and susceptibility to HF [104]. TRPC6 is upregulated in Wistar rat hearts by high-salt diet-induced inflammation and is associated with the increase in fibrosis and hypertension as a result of excessive salt consumption [102]. Mouse cardiac TRPC6 is also upregulated in the whole heart in response to STZ-induced hyperglycaemia, which destabilised the formation of a TRPC3-Nox2 complex, counteracting STZ-induced oxidative stress [90]. Moreover, co-genetic depletion of TRPC3 and TRPC6, but not TRPC6 alone, greatly improved outcomes following LV PO in mice [106]. It is therefore evident that TRPC6 plays a complex but important role in cardiopathology, and that expanding current understanding of the role TRPC6 plays in each type of cardiac cell population would be essential for delineating the function of the channel in HF progression.

In summary, the role of TRPC6 in regulating myocardial remodelling can be largely viewed as being profibrotic, but whether this is beneficial or detrimental depends on the particular pathology studied. The heteromeric nature of TRPC channels makes interpretation of the role of TRPC6 in isolation difficult to evaluate.

#### 2.1.2. TRPC6 in Cardiac Fibroblasts

The concept that TRPC6 promotes cardiac fibrosis during cardiac dysfunction is strongly supported by in vitro analysis of cultured CF [101,103,104]. A genome-wide screen identified TRPC6 expression as being required for MF differentiation, with treatment of cultured rat CF with TGF-β or Ang II inducing a concentration-dependent increase in TRPC6 expression [101]. Overexpression of the channel prompted spontaneous differentiation of fibroblasts to MF, while TRPC6 KO attenuated MF differentiation induced by TGF-β (in mouse dermal fibroblasts) and Ang II (in rat CF) [101]. However, it should be noted that all TRPC channels have lately been described as being dispensable for Ang II-evoked Ca^2+^ entry in CF, with the CRAC channel Orai1 driving the pathological Ca^2+^ in response to Ang II stimulation [107]. This suggests the correlation between TRPC6 upregulation may occur as a consequence of pathological rises in intracellular Ca^2+^, but is likely not the driver of Ca^2+^ influx.

In rodents, TRPC6 further promoted CF differentiation via a TGF-β/p38 MAPK/TRPC6/calcineurin–NFAT signalling pathway [101]. TGF-β induced p38 MAPK activation and upregulated TRPC6 through driving serum response factor transcriptional regulation of the TRPC6 promoter region, and subsequent upregulation of TRPC6 gene expression [101]. TGF-β/p38 MAPK-driven TRPC6 activation and increased Ca^2+^ permeability in cultured rat CF induced MF transformation via calcineurin–NFAT signalling [101]. This signalling module has been further demonstrated to drive fibrosis during right ventricular PO, in an endoglin-dependent mechanism [103].

Together, these over-expression and KO studies suggest that TRPC6 is important for differentiation of CF to MF (Figure 2B). Whether TRPC6 is acting as a primary mechanosensor, or is upregulated as a consequence of Ca^2+^ entry via other channels (e.g., Orai), requires more focused exploration.

### 2.2. Melastatin Family of Transient Receptor Potential (TRPM) Channels

The TRPM subfamily comprises eight channels (TRPM1–TRPM8) expressed in mammals. These channels differ in tissue distribution, cation selectivity, and activating mechanisms, and have been implicated in a diverse array of cellular functions, including cell proliferation, cell invasion, temperature sensing, magnesium homeostasis and taste [86,108]. Dysregulation of some TRPM channels contributes to cancer promotion, cerebral ischemia-reperfusion injury and cardiac fibrosis [108,109,110,111,112,113]. Within the heart, transcriptomic analysis of mouse cardiac tissue has identified only TRPM4 and TRPM7 as being expressed within the atrial myocardium, while TRPM1, 3, 4, 6 and 7 are expressed within the ventricular myocardium [114]. Expression of both TRPM2 and TRPM7 has been detected in CF, despite an apparent lack of expression of the former in mouse myocardium [115,116]. However, TRPM2 is not a mechanosensitive ion channel, but is activated by ADP-ribose binding [117].

#### 2.2.1. TRPM7

**Figure 3:**
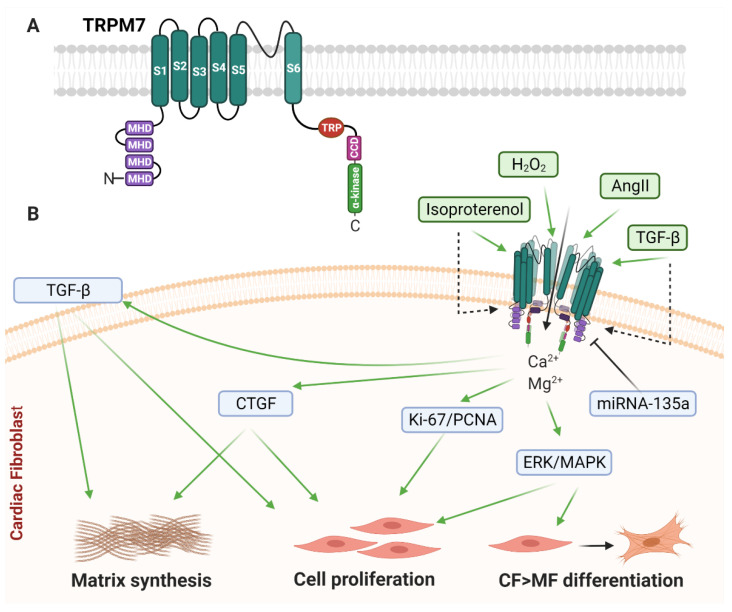
Schematic diagram of TRPM7 structure and cardiac fibroblast signalling. (**A**) Basic structure of alpha subunit of TRPM7. Each TRPM7 alpha subunit is comprised of six transmembrane-spanning domains (S1–S6), with a pore loop between S5 and S6, melastatin homology domains (MHD) at the amino termini, and a TRP-box (TRP), a coiled-coil domain (CCD), and an atypical α-type serine/threonine protein kinase domain (α-kinase) at the carboxyl termini. (**B**) Known TRPM7 homotetrameric ion channel signalling in cardiac fibroblasts. Ang II drives activation of TRPM7 and is coupled to increased nuclear activity of Ki-67 and proliferating cell nuclear antigen (PCNA), as well as upregulation of markers of fibrosis including connective tissue growth factor (CTGF). H_2_O_2_ activates TRPM7 and promotes MF differentiation and ECM remodelling via the ERK-1/2 MAP kinase pathway. TGF-β1 and isoproterenol treatment both induce upregulation of TRPM7 expression and Ca^2+^ influx leading to myofibroblast differentiation. TRPM7 expression can be inhibited by miR-135a expression. See main text for details.

TRPM7 (Figure 3A) is a ubiquitously expressed cation channel permeable to Mg^2+^, Ca^2+^ and Zn^2+^ under physiological conditions, with permeation of Mg^2+^ acting as a negative feedback mechanism [118]. The C-terminus of TRPM7 acts as an α-kinase, and as such has been referred to as a “chanzyme” due to its function as a cation channel with enzymatic kinase activity. The cytoplasmic C-terminus is rich in serine/threonine residues and is capable of auto-phosphorylation and phosphorylation of downstream targets [119,120].

TRPM7 forms tetrameric channels which are either homomeric, or heteromultimeric with TRPM6, with each type of channel configuration having distinct functions. Both TRPM6 and TRPM7 channels are vital for Mg^2+^ homeostasis [118].

TRPM7 can be regulated through multiple mechanisms that influence cardiac function. PIP_2_ has been implicated as an endogenous inhibitor of TRPM7 in rat CM and CF following receptor-mediated PLC activation [121]. Although the patch clamp-detected currents were attributed to TRPM7, this was not confirmed, for example using TRPM7-deficient cells [121]. Aldosterone treatment of TRPM7-expressing HEK 293 cells increased expression and plasma membrane localization of TRPM7 [122]. In human and rat vascular SMC, bradykinin treatment increased phosphorylation of serine/threonine residues on the TRPM7 C-terminus and increased TRPM7-dependent Mg^2+^ influx [123]. However, it should be noted that mutations in TRPM7 residues in either the channel or kinase domain do not influence the activity of one another, and thus the two likely function independently of one another [124].

TRPM7 is responsive to membrane stretch and shear flow in various exogenous and endogenous expression systems [75,76,125,126,127,128]. For example, heterologously expressed TRPM7 is responsive to membrane stretch (suction) and osmotic swelling in HeLa cells [75] and osmotic swelling in HEK 293 cells [76] (Table 1). Changes to TRPM7 single-channel activity were detected using cell free patch (excised inside-out) and whole cell patch [76]. Within MDA-MB-231 breast adenocarcinoma and HT1080 fibrosarcoma cells, TRPM7 acts as a mechanosensor of hydraulic resistance, which drives Ca^2+^ influx; a function which is abolished by TRPM7 inhibition or functional TRPM7 KO [127].

In each example of TRPM7 mechanosensitivity, the TRPM7-driven increase in intracellular Ca^2+^ was abolished by depletion of extracellular Ca^2+^ [75,76,125,127]. Thus, evidence supporting TRPM7 as being responsive to mechanical stress is robust, however it does not confirm that TRPM7 is inherently mechanosensitive, as the mechano-response of TRPM7 has not been demonstrated independently of other cellular components.

#### 2.2.2. TRPM7 and Cardiac Remodelling

The role of TRPM7 in the heart is complex; within rodent models the activity of TRPM7 has been described to act both in promoting pathological signalling mechanisms [112], as well as in cardioprotective mechanisms [129,130]. In relation to the heart, TRPM7 expression in humans, rodents, and zebra fish has been detected in myocytes, the sinus node, and both atrial and ventricular fibroblasts [109,121,131,132], as well as infiltrating immune cells in mice [129]. TRPM7’s cardioprotective function may principally be due to homeostatic regulation of cellular Mg^2+^ rather than Ca^2+^ levels [130]; while TRPM7 regulation of Ca^2+^ levels have been implicated in cardiac fibrosis [109,110,111,133].

Rio et al. reported that the kinase domain of TRPM7 is important for opposing cardiac fibrosis in mice [129]. Mice with global TRPM7 deficiency, or TRPM7 kinase domain deletion (TRPM7^+/Δkinase^ mice), were significantly more prone to cardiac hypertrophy, fibrosis, and inflammation [129]. Expression of many markers of cardiac fibrosis and inflammation were strongly upregulated in the TRPM7^+/Δkinase^ mice, including expression of profibrotic molecules, structural ECM proteins and inflammatory cytokines [129]. Interestingly, this cardioprotective role of TRPM7 kinase domain was attributed to TRPM7 function within infiltrating macrophage cells rather than CF [129].

Conversely, Lu et al. suggest TRPM7 expression is associated with an increase in hypoxia-induced fibrosis [112]. The expression levels of TRPM7 in CF isolated from neonatal Sprague Dawley rat hearts, detected at the protein level, appeared to be slightly upregulated during hypoxia-induced cardiac fibrosis [112]. Whole-cell patch recording of isolated rat CF exposed to hypoxia, indicated an increase in whole-cell currents [112]. However, it should be noted that other mechanosensitive cation channels (e.g., Piezo1—see Section 3) [134] are also expressed in CF, so this should be taken into consideration when interpreting the changes in whole-cell patch recordings. The upregulation of TRPM7 in CF, due to hypoxia, correlated with an increase in cardiac fibrosis [112].

In summary, the overall role of TRPM7 in cardiac remodelling is complicated by the different functional coupling of Ca^2+^ and Mg^2+^ ions, and the expression of the channel in multiple cardiac and immune cell types. Thus, TRPM7 may differentially influence cardiac pathology, depending on which cell type has functionally active TRPM7, and the environmental differences underlying channel functionality.

#### 2.2.3. TRPM7 in Cardiac Fibroblasts

Current research indicates that TRPM7 expression promotes CF proliferation and differentiation. TGF-β1 in human CF, [109] and Ang II, hydrogen peroxide, hypoxia, and isoproterenol treatment in rat hearts [110,111,112,113] all promote TRPM7 activation and subsequent increases in α-SMA and collagen synthesis in CF. However, TRPM7 has yet to be demonstrated to respond to mechanical stimuli in driving the MF phenotype.

In mediating CF responses to Ang II in rats, TRPM7 is potentially important in perpetuating a pathological phenotype [110,135]. Ang II-driven TRPM7 activation, and subsequent CF proliferation, evoked increases in cell cycle-related regulatory protein Ki-67 and proliferating cell nuclear antigen (PCNA), as well as markers of fibrosis including α-SMA and collagens type I and III [110]. SiRNA knockdown of TRPM7 reduced Ang II-evoked expression of Ki-67, PCNA and α-SMA, while also attenuating Ang II-driven TRPM7-evoked currents, detected using whole-cell patch clamping [110]. In further support of a role for TRPM7 in Ang II signalling, Ang II treatment of mouse CF increased TRPM7 protein expression [133]. Yang et al. reported that TRPM7-induced Ca^2+^ and Mg^2+^ influx was required for Ang II-driven CF proliferation and upregulation of markers of fibrosis [133]. Treatment of cultured mouse CF with the TRPM7 inhibitor 2-aminoethoxydiphenylborate (2-APB) attenuated Ang II-induced upregulation of connective tissue growth factor (CTGF) and α-SMA, and inhibited CF proliferation [133]. However, it should be noted that 2-APB also activates other mechanosensitive ion channels, including K_2P_ channels [136] and other TRP channels [137].

To further implicate fibroblast TRPM7 function in pathological cardiac remodelling, TRPM7 upregulation correlated with TGF-β1 stimulation in cultured human atrial fibroblasts, leading to an increase in Ca^2+^ influx and MF differentiation [109]. H_2_O_2_-induced TRPM7 activity promoted MF differentiation through increasing intracellular Ca^2+^ and activation of the ERK-1/2 MAP Kinase signalling pathway in rats [111]. In vitro chemical and genetic inhibition of rat TRPM7 with 2-APB and shRNA, respectively, inhibited H_2_O_2_-induced Ca^2+^ influx and ERK-1/2 phosphorylation, and attenuated the increased expression of fibrotic markers including collagen type I, fibronectin, α-SMA, CTGF, and TGF-β1 [111]. Further to this, TRPM7 was upregulated in rat CF following subcutaneous administration of isoproterenol treatment, also resulting in upregulation of α-SMA and collagen type 1 [113]. Interestingly, the expression of TRPM7 has been shown to be inversely correlated with miRNA-135a expression; increasing levels of TRPM7 expression in rat CF corresponded to decreased levels of miRNA-135a, while the over-expression of miRNA-135a led to a marked decrease in TRPM7 [113,138]. In vitro inhibition of TRPM7, through either siRNA knockdown or miRNA-135a mimetics, attenuated the increase in α-SMA and collagen type 1 [113,138].

Taken together, these studies reveal a clear consensus that TRPM7 drives numerous profibrotic changes at the level of the CF, including cell proliferation, MF differentiation, ECM protein synthesis and profibrotic paracrine signalling (Figure 3B), which likely underlie several of the pathological effects of this channel in cardiac remodelling. However, it should be noted that there is currently no evidence that mechanical stimulation of TRPM7 can induce such changes in CF.

### 2.3. Vanilloid Family of Transient Receptor Potential (TRPV) Channels

The TRP Vanilloid family comprises 6 channels (TRPV1-6) that are primarily associated with sensitivity to noxious temperature [93], although TRPV channels have also been described to respond to chemical ligands, osmotic stress, and mechanical force [93]. TRPV1, 2, 3 and 4 are all expressed in the mammalian myocardium [139]. However, neither TRPV2 nor TRPV3 are functional within CF [140,141].

#### 2.3.1. TRPV1

**Figure 4:**
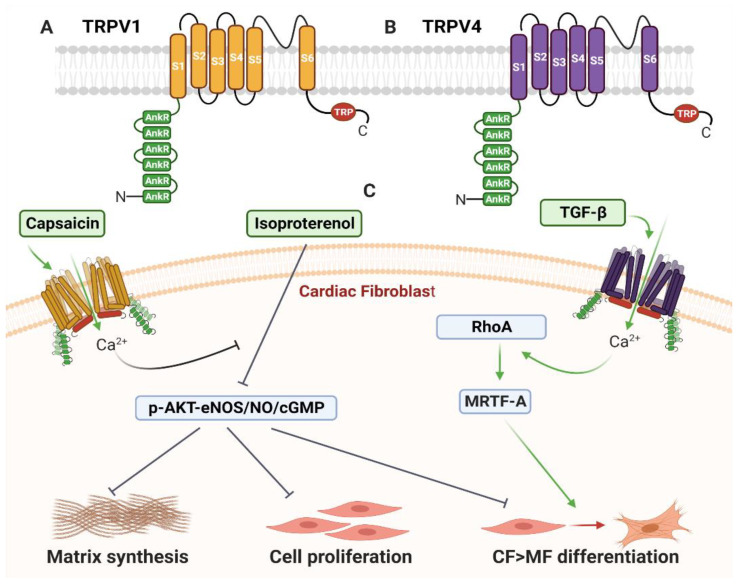
Schematic diagram of TRPV1 and TRPV4 structure and cardiac fibroblast signalling. (**A**) Basic structure of alpha subunit of TRPV1. (**B**) Basic structure of alpha subunit of TRPV4. (A-B) Each TRPV1 and TRPV4 alpha subunit is comprised of six transmembrane-spanning domains (S1–S6), with a pore loop between S5 and S6, ankyrin repeats (AnkR) at the amino terminus, and a TRP-box at the carboxyl terminus. (**C**) Known TRPV1 and TRPV4 homotetrameric ion channel signalling in cardiac fibroblasts. Capsaicin activates TRPV1 and inhibits Ang II-induced cell proliferation. TRPV1 activation also inhibits isoproterenol-induced cell proliferation, and attenuates isoproterenol-induced ECM synthesis, cell proliferation and myofibroblast differentiation by blocking downregulation of p-Akt, p-eNOS, NO and cGMP. TGF-β activates TRPV4, inducing Ca^2+^ influx and promoting RhoA signalling, upregulation of MRTF-A transcription factor, and myofibroblast differentiation. See main text for details.

TRPV1 (Figure 4A) is considered to be the most widely studied of the TRPV family and in mammals is primarily expressed in sensory nerve fibres whereby cation influx stimulates action potentials and neurotransmitter release [142,143]. TRPV1 is a non-selective cation channel, which enables movement of monovalent cations, with a ten-fold preference for Ca^2+^ [142]. With respect to the heart, TRPV1 expression has been detected in a range of cells isolated from mouse hearts, including afferent fibres [144], myocytes [145], endothelial cells (EC) and SMC [146], as well as fibroblasts [147].

TRPV1 is activated, or sensitised to activation, by the binding of the vanilloid capsaicin, and various lipids including arachidonic acid metabolites such as 12(S)-hydroxyglutaric acid, 12-hydroxyhexanedienic acid and 20-hydroxyeicosatetraenoic acid (20-HETE) [146]. TRPV1 may also become active in response to nociceptive thermal stimulation and low pH [148]. TRPV1 functionality is regulated by PIP2 and PLC; with the binding of TRPV1 to PIP2 holding the channel within an inactive state, that can be released by PLC-mediated PIP2 hydrolysis [148].

In mammals, TRPV1 acts as a mechanosensor in response to multiple forms of mechanical stimuli. For example, TRPV1 acts as an osmoreceptor in response to hypotonicity [149], a thermo-sensor [148], and as an intravascular mechanosensor for changes in blood pressure [150], and in the bladder and digestive system [151,152,153,154].

Despite overwhelming evidence of TRPV1’s ability to activate in response to mechanical stimulation, it may not directly detect mechanical stretch of the membrane. Single-channel conductance measurements of mammalian TRPV1, using patch clamping applied pipette pressure, failed to detect TRPV1 channel generated currents, suggesting the channel is not stretch sensitive when over-expressed in HEK 293 cells [65] (Table 1). In male Sprague Dawley rats, the ability of TRPV1 to induce modulation to intraluminal pressure has been suggested to be dependent on the accumulation of the arachidonic acid metabolite 20-HETE, prior to channel activation [155]. Interestingly, Borbiro et al. found TRPV1 inhibited Piezo1 function in mouse dorsal root ganglion neurons, through depleting membrane phosphoinositides [156]. Together, these data suggest an important, but indirect, role for TRPV1 in modifying the cellular response to membrane stretch. A more comprehensive review of TRPV1 can be found in [142].

#### 2.3.2. TRPV1 and Cardiac Remodelling

TRPV1 functionality within the heart is primarily thought to protect against cardiac dysfunction and adverse remodelling. For example, TRPV1 is important for pre-conditioning protection against MI in rodents [157,158,159,160]. Moreover, in rodent models of MI, TRPV1 may act in suppressing the inflammatory and pathological cardiac remodelling response in the infarct region following ischemic injury [161,162]. Mice with global TRPV1 depletion exhibited an increase in infarct size and mortality after LAD coronary ligation, with a significantly higher level of MF infiltration, capillary density and collagen content [162]. Depletion of TRPV1 in mice also increased TGF-β, Smad2, VEGF and MMP2 expression [162] and significantly reduced expression of the cardioprotective protein calcitonin gene-related peptide (CGRP) and substance P within the myocardium [160,161]. Downregulation of TRPV1, CGRP and substance P in the whole heart of diabetic rats has been implicated in loss of cardioprotection during the progression of diabetes [160].

Studies employing the TAC model of LV PO have further confirmed the protective role of TRPV1 during adverse cardiac remodelling and fibrosis [163,164]. TRPV1 global KO mice which had undergone TAC experienced an increase in hypertrophy, collagen deposition and infiltration of immune cells compared to WT animals [163,164]. In further support of the cardioprotective role for TRPV1, mice fed chow containing the TRPV1 agonist capsaicin experienced less adverse effects of PO, including reduced cardiac hypertrophy and fibrosis, compared with mice fed regular chow [147]. Moreover, long-term dietary intake of capsaicin also protected against high salt diet-induced adverse cardiac remodelling; a protective effect that was not detected in TRPV1 KO mice [145,165].

Given that TRPV1 is expressed in many different cell types within the heart, and functions in response to a diverse array of stimuli, studies of whole hearts can mask how the channel functions within individual cell types during the progression of cardiac dysfunction. This concept is supported by a recent study in a transgenic mouse model with TRPV1 depleted specifically in afferent neurons, identifying activation of TRPV1 within afferent neurons as promoting fibrosis and adverse cardiac remodelling following MI [166].

In summary, there is good evidence that TRPV1 activity is protective for cardiac remodelling. However, characterization of cell type-specific TRPV1 (potentially through targeted inducible transgenic mouse models) will be necessary to gain a more complete understanding of how this channel acts at the cellular level to regulate cardiac dysfunction.

#### 2.3.3. TRPV1 in Cardiac Fibroblasts

The majority of studies aimed at understanding the function of TRPV1 within the heart have analysed whole-heart TRPV1 channel activity, and KO mouse models have mostly been global deletions of TRPV1; thus little is known of how TRPV1 influences CF function specifically in vivo. However, Wang et al. reported that murine CF express functional TRPV1 channels, as CF were sensitive to the TRPV1 channel agonist, capsaicin [147]. In vitro analysis of cultured CF indicated capsaicin mitigates Ang II-induced CF proliferation in cells from WT mice but not TRPV1 KO mice; suggesting TRPV1 is functional within CF, and coupled to inhibition of fibroblast proliferation [147]. Furthermore, cultured CF isolated from transgenic mice overexpressing TRPV1 are resistant to isoproterenol-induced MF differentiation [167]. Over-expression of TRPV1 suppressed isoproterenol-induced proliferation and attenuated isoproterenol-induced increases in expression of collagen type 1, collagen type 3 and fibronectin, while partially blocking downregulation of p-Akt and p-eNOS, and the decrease in NO and cGMP in mouse CF [167]. The TRPV1-dependent inhibition of the MF phenotype was reversed by treatment of cells with the eNOS inhibitor L-NAME, which prevented the TRPV1-stimulated increase in NO and cGMP [167]. In rodents, eNOS has cardioprotective effects following MI, via suppression of ROS formation and subsequent oxidative stress-induced TGF-β expression [168,169,170]. Hence, Wang et al. suggested that CF TRPV1 function opposes fibrosis through enabling Ca^2+^ influx and subsequent regulation of the eNOS/NO pathway [167].

In summary, the specific effects of TRPV1 on CF indicate that many of its protective effects on myocardial remodelling may be due to inhibition of specific CF functions, including cell proliferation, MF differentiation and ECM protein synthesis via activation of an eNOS/NO/cGMP-dependent pathway (Figure 4C).

### 2.4. TRPV4

TRPV4 (Figure 4B) is a Ca^2+^- and Mg^2+^-permeable channel that is activated in response to heat, osmotic swelling, mechanical force, binding of the arachidonic acid metabolite 5′,6′-epoxyeicosatrienoic acid, and phorbol ester compounds [65,77,171]. Whether TRPV4 is truly mechanosensitive is uncertain, and complicated through conflicting reports that describe a functional role for TRPV4 in some forms of mechanosensing. Single-channel conductance measurements of mammalian TRPV4, using patch clamping applied pipette pressure, determined that TRPV4 channels were not stretch sensitive when over-expressed in HEK293 cells [65] (Table 1). Servin-Vences and colleagues also found TRPV4 in murine chondrocytes was not activated by membrane stretch; however, application of mechanical force at points of contact between cells, and the ECM, appeared to generate TRPV4-dependent electrical currents [67].

Some of these effects may be due to TRPV4 acting downstream of other mechanosensitive channels, such as Piezo1 (see Section 3) [77,172]. For example, Swain and colleagues reported that Piezo1 and TRPV4 act in concert to initiate and sustain Ca^2+^ influx in response to mechanical stimulation in human pancreatic acinar cells [77] and in response to shear fluid stress in human EC [172], with activation of Piezo1 prompting TRPV4 activity. TRPV4 knockdown also diminished Piezo1 sensitivity to Piezo1-specific agonists [77], thus indicating that the channels may act in concert to confer mechanical signalling, with Piezo1 acting as the mechanical force sensor [77,172].

With this in mind, the following discussion of TRPV4 function in cardiac and CF biology should be interpreted with consideration for the types of chemical and mechanical forces the channel is known to respond to, in addition to other proteins that may act in concert with TRPV4 in generating a response.

#### 2.4.1. TRPV4 and Cardiac Remodelling

TRPV4 is expressed within all mammals [173] and has been detected in multiple cardiac cell types, including fibroblasts, EC [174] and CM [175,176,177,178]. Murine global TRPV4 deletion improves survival rates and preserves ejection fraction in mice that have undergone TAC or MI surgery [179]. TRPV4 KO mice also had significantly less cardiac fibrosis post TAC or MI surgery, with reduced expression of profibrotic markers Col1a2, α-SMA, N-FAT, TGF-β1, and the mechanosensitive transcription factor MRTF-A in whole-heart tissue [179]. TRPV4 global KO have also been shown to exhibit decreased CM hypertrophy and CF differentiation 28-days post TAC surgery [180]. Moreover, oral treatment of TRPV4 KO mice with the TRPV4 antagonist, GSK2193874, further confirmed the cardioprotective effect of TRPV4 inhibition [181]. It should also be noted that a preceding study from the same research group attributed some of the pathological cardiac functions of TRPV4 to endothelial-specific TRPV4 activity [182].

Cardiac TRPV4 is upregulated in the myocardium of diabetic rats and has been implicated in driving diabetes-induced cardiac fibrosis [183]. In vivo chemical inhibition of TRPV4 in cultured diabetic rat CF, with the antagonist HC067047, reduced expression of markers of MF differentiation and attenuated increases in TGF-β1 levels, while also reducing phosphorylation of Smad3 [183].

Taken together, these studies indicate that TRPV4 plays an important role in inducing pathological myocardial remodelling, including cardiac hypertrophy and fibrosis.

#### 2.4.2. TRPV4 in Cardiac Fibroblasts

CF undergo Ca^2+^ influx in response to the TRPV4-specific agonist 4α-phorbol 12, 13-didecanoate [184]. In vitro analysis of mouse CF, isolated from TRPV4 KO mice, indicated TRPV4 activates in response to TGF-β1, and drives RhoA signalling and an increase in expression of the mechanosensitive transcription factor MRTF-A [180]. Within the same study, it was demonstrated that inhibition of MRTF-A attenuates TGF-β1-induced CF differentiation. Moreover, cultured CF isolated from Sprague Dawley rats and treated with TRPV4-targeting siRNA, or CF isolated from TRPV4 KO mice, did not differentiate into MF following TGF-β1 treatment [185,186]. Importantly, CF isolated from TRPV4 KO mice were resistant to differentiation in response to high-stiffness ECM gels and hypotonicity-induced Ca^2+^ influx; and the attenuation of differentiation was not reversed by saturating amounts of TGF-β1 [186].

Together, these studies suggest a key role for TRPV4 in coupling mechanical stimulation and TGF-β1 signalling to adoption of the fibrotic MF phenotype through a RhoA/MRTF-A and Smad3 signalling mechanism (Figure 4C).

## 3. Piezo1 Channel

The field of mechanosensitive ion channels has been revolutionised by the recent discovery of Piezo1 and Piezo2 non-selective cation channels that are inherently mechanosensitive and act as primary force sensors in a wide range of mammalian cells and tissues [187,188,189]. The Piezo1 ion pore is selective for divalent (Ba^2+^, Ca^2+^, Mg^2+^ and Mn^2+^) and monovalent (K^+^, Na^+^, Cs^+^ and Li^+^) cations [190]. In contrast to Piezo2 channels that are primarily expressed in sensory tissues, Piezo1 is widely expressed in cells and tissues responsible for regulating many aspects of the cardiovascular system [191]. For example, endothelial Piezo1 acts as a sensor of shear stress, and is required for formation and regulation of vascular structure in developmental and adult physiology [192,193,194,195], and as an important regulator in sensing blood pressure and the baroreceptor reflex [196]. Within the heart, Piezo1 is expressed in both myocytes and fibroblasts, and is essential for cardiac outflow tract and aortic valve development [197,198], the response to cyclic stretch [199], and for mediating homeostatic cardiac mechano-chemical transduction [200]. Furthermore, Piezo1 expression in arterial SMC influences remodelling of small arteries during hypertension [201]. For a more in-depth review of Piezo1 in cardiovascular health and disease, please refer to this recent article [191].

**Figure 5:**
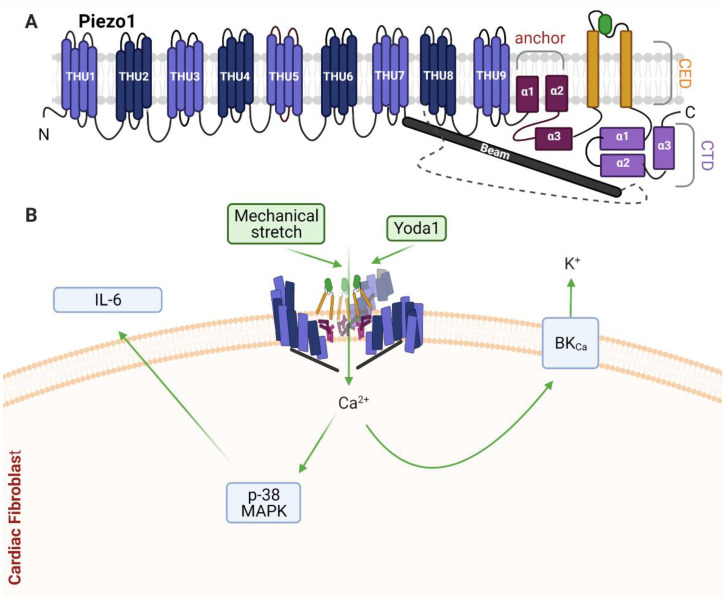
Schematic diagram of Piezo1 structure and cardiac fibroblast signalling. (**A**) Predicted basic alpha subunit structure of mammalian Piezo1. Piezo1 is predicted to have a peripheral transmembrane segment comprised of nine transmembrane helical units (THU1-THU9), an anchor domain (α1-3), C-terminal extracellular domain (CED), C-terminal domain (CTD), and a beam-like structure facing the intracellular surface. (**B**) Known Piezo1 homotrimeric ion channel signalling in cardiac fibroblasts. Mechanical stretch and Yoda1 both activate Piezo1 in cardiac fibroblasts. Mechanical stimulation of Piezo1 activates the potassium-selective Ca^2+^-activated channel of large conductance (BK_Ca_). Yoda1 activation of Piezo1 activates the p38α MAPK pathway and stimulates IL-6 gene expression and protein secretion. See main text for details.

The unique structure of the Piezo channels (Figure 5A) enables them to directly sense membrane tension [78,202] (Table 1). The Piezo1 channel comprises three Piezo1 proteins arranged to form a trimer, or triskelion shape [202,203,204,205,206], with three propeller blade-like structures projecting outward from a central pore, formed at the C-terminus of each protein [207]. The central pore constitutes the non-selective cation channel [207], with an alpha helix beam connecting each propeller-like blade structure to the central pore [203,208]. Piezo1 has a curved structure, creating a dome shape in the membrane [204] which extends beyond the radius of Piezo1 [208]. Flattening of the dome shape, via mechanical tension, has been hypothesised to provide the energy requirement for mechanical activation of the channel [208].

Piezo1 responds to a number of different types of mechanical stimuli in tissue culture, including lateral membrane tension [79,202,209], compression [210,211], osmotic swelling [212], and rhythmic mechanical stimulus [213], including 24-h cyclic stretch [214]. However, it should be noted that Piezo1 responsiveness to cyclic-stretch appears to be cell type-specific [34]. Substrate stiffness can also modulate Piezo1 activity in vitro; an increase in substrate stiffness reduced Piezo1 channel activation in HEK 293T cells transiently transfected with human Piezo1 [215]. Conversely, a reduction in the density of contact points between a cell and the external substrate, represented as roughness of the substrate, increased Piezo1 channel activity in HEK 293T cells expressing human Piezo1 [215]. Bavi and colleagues suggested that the responsiveness of Piezo1 to substrate stiffness and roughness indicates a synergistic mechanism between force sensed by the phospholipids and the actin cytoskeleton in HEK 293T cells expressing human Piezo1 [215], a concept further supported by evidence that Piezo1 acts as a novel component of integrin-based adhesions [34]. It should be noted that the latter reference [34] is a pre-print that has not yet been peer-reviewed. Together, studies elucidating the activation mechanisms of Piezo1 suggest the channel is involved in integration of many forces that influence the tension of the plasma membrane and external environment surrounding the cell. Piezo1 can also be activated by the synthetic small molecule Yoda1 [216], which serves as a useful tool for chemical activation of the channel in in vitro experiments.

Following channel activation, Piezo channels can rapidly enter a non-conducting state to become inactivated [78]. Lewis and Grandl established that Piezo1 can be inactivated by resting membrane tension [79]. The resting force of the plasma membrane may be enough to drive channel inactivation and prevent reactivation of Piezo1, indicating a highly sensitive tuning mechanism for Piezo1 in response to membrane tension [79]. The inactivation state of Piezo1 may also be influenced by divalent ion concentration [217], fatty acid composition within the plasma membrane [218], and protonation of the channel [219].

### 3.1. Piezo1 and Cardiac Remodelling

Surprisingly little is known about Piezo1 function in the heart, likely reflecting the relatively recent identification of this channel. Nonetheless, Piezo1 has been shown to be expressed in several cardiac cell types, including CM, CF and EC, in both humans and mice [134], with higher expression in fibroblasts than myocytes [134,220].

Piezo1 was shown to be upregulated in the hearts of male Sprague Dawley rats following experimental MI, and in vitro studies indicated upregulation of Piezo1 via an Ang II-ERK1/2-dependent pathway in CM [221]. However, whether the observed increase in Piezo1 expression in the heart occurred within the myocyte or non-myocyte cell population was not evaluated. Furthermore, the significance of the increase in cardiac Piezo1 expression was not explored, for example using genetic Piezo1 KO. Very recently, a more comprehensive study has suggested a key role for myocyte Piezo1 in regulating homeostatic mechano-chemical signalling in the heart [200]. Murine studies revealed that cardiac-specific Piezo1 deletion led to impaired heart pump function, whereas cardiac-specific overexpression of Piezo1 led to severe HF and arrhythmia; together indicating an essential role for myocyte Piezo1 in maintaining normal cardiac function [200]. Doxorubicin-induced dilated cardiomyopathy induced an increase in Piezo1 expression in CM, and clinical data revealed increased Piezo1 expression in heart biopsies from patients with hypertrophic cardiomyopathy [200].

In summary, a small number of studies are now suggesting that Piezo1 acts as a novel mechanosensor in the heart, and that its expression is increased in cardiac pathologies. More studies are eagerly anticipated to further define the role of Piezo1 in myocardial remodelling.

### 3.2. Piezo1 in Cardiac Fibroblasts

We recently reported that Piezo1 is expressed and functionally active in human and mouse CF [134]. Cell-attached patch clamp recordings established that human CF (specifically atrial fibroblasts) contain pressure-induced mechanically activated ion channels whose activity was reduced by Piezo1 siRNA knockdown. Moreover, the Piezo1 agonist Yoda1 stimulated Ca^2+^ entry in mouse and human CF, which was inhibited by pharmacological Piezo1 blockers, Piezo1 gene silencing or Piezo1 KO [134]. Piezo1 activation by Yoda1 was coupled to activation of the p38α MAPK pathway and IL-6 gene expression and protein secretion [134]. Furthermore, basal IL-6 production in CF cultured on softer collagen-coated substrates was reduced by Piezo1 siRNA knockdown, suggesting culture conditions (matrix composition and stiffness) may alter Piezo1 signalling in vitro. Increased circulating IL-6 concentrations have been associated with cardiac fibrosis and promotion of hypertension [222,223,224]. Within the heart, IL-6 may influence regulation of the ratio of cell populations, composition of the ECM, and cell–cell interactions including crosstalk between myocytes and fibroblasts [225]. Indeed, IL-6 may be important as a paracrine signalling molecule released from CF to induce CM hypertrophy [226]. The p38α pathway in fibroblasts is emerging as a critical regulator of cardiac remodelling [224] and fibroblast-specific p38α KO mouse models have revealed important roles for this kinase in driving both cardiac hypertrophy [226] and fibrosis [227].

In a very recent pre-print report [69], and in agreement with our study [134], mechano-activation of Piezo1 was confirmed in human atrial fibroblasts via cell-attached patch clamp recordings following application of negative pressure. Interestingly, CF isolated from atrial fibrillation patients expressed a higher level of Piezo1 expression and exhibited an increase in Piezo1 activity when compared to cells from sinus rhythm patients [69], indicating a potentially important role in atrial fibrillation and remodelling. Mechanical stimulation also activated the potassium-selective Ca^2+^-activated channel of large conductance (BK_Ca_) in these cells. While Piezo1 was directly activated by stretch, mechanical induction of BKCa activity was secondary to calcium influx via Piezo1, suggesting interplay between these channels in human atrial fibroblasts [69]. Another recent report from the same group described a role for Piezo1 in increasing atrial fibroblast cell stiffness in response to increased ECM stiffness [228]. Over-expression and siRNA studies revealed that Piezo1 positively affected stiffness of a human atrial fibroblast cell line, including increasing the amount, thickness and organization of the F-actin network. Moreover, Piezo1 over-expressing cells could confer increased stiffness to neighbouring non-transfected cells through a paracrine IL-6 signalling mechanism [228]; similar to that identified in our recent study [134].

Together, these recent in vitro studies highlight the emerging significance of Piezo1 function in CF (Figure 5B). Future studies to address mechanical signalling, and to evaluate the role of fibroblast-specific Piezo1 in modulating cardiac physiology and pathological cardiac remodelling in vivo, will be important to more clearly define its function in the heart.

## 4. TWIK-Related Potassium Channel-1 (TREK-1)

**Figure 6:**
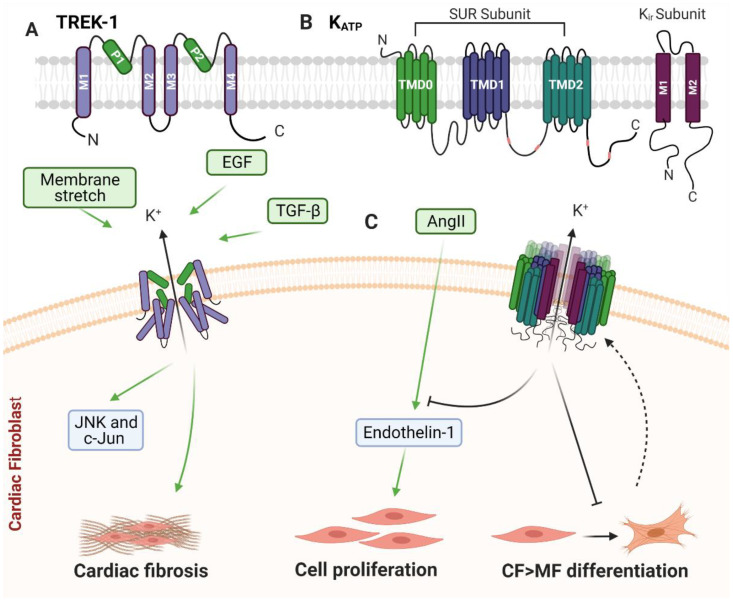
Schematic diagram of TREK-1 and K_ATP_ structure and cardiac fibroblast signalling. (**A**) Predicted basic alpha subunit structure of TREK-1. TREK-1 subunits are composed of four transmembrane domains (M1-M4) and two pore forming P-loops (P1-P2). (**B**) Predicted basic alpha subunit structure of K_ATP_. Each K_ATP_ alpha subunit consists of one ABC transporter subunit (SUR) and one inward rectifier K^+^ channel (Kir) subunit (**C**) Known TREK-1 homodimeric ion channel and K_ATP_ tetrameric ion channel signalling in cardiac fibroblasts. Cardiac fibroblast TREK-1 activation promotes cardiac fibrosis. Membrane stretch, TGF-β and EGF modulate JNK and c-Jun phosphorylation via a TREK-1-dependent mechanism. K_ATP_ gene expression is upregulated during cardiac fibroblast to myofibroblast transdifferentiation. Once active, K_ATP_ attenuates Ang II-induced proliferation and endothelin-1 expression, while also inhibiting myofibroblast differentiation. See main text for details.

TREK-1 channels (Figure 6A) are one of fifteen types of two-pore domain potassium channels (K_2P_) expressed in human cells [229]. K_2P_ are classed as background, or leak, transmembrane K^+^ channels and are associated with maintaining resting membrane potential and reducing action potential firing through cellular potassium regulation in mammals [230,231]. K_2P_ channels may also act as counterparts to Piezo1’s non-selective ion channel depolarization activity, enabling fine-tuning of mechanosensation [232]. Of the fifteen different types of K_2P_ channels, only TWIK-related K1 (TREK-1), TWIK-related K2 (TREK-2) and TWIK-related arachidonic acid-activated K^+^ channel (TRAAK) have been described as mechanosensitive ion channels [233]. However, to date, only TREK-1 has been identified as expressed and functional in CF [234].

In contractile tissues and organs in mammals, TREK-1’s function in cellular hyperpolarization may reduce cellular contraction in response to mechanical forces [232]. K_2P_ channels lack a voltage sensing domain. However, TREK-1 has been described as voltage responsive due to an ion-flux gating mechanism [235]. The voltage dependency of TREK-1 is activated by the bio-active lipids arachidonic acid and PIP_2_ [235,236,237]. TREK-1 can be activated by membrane receptor-coupled second messengers, heat, intracellular acidosis and volatile anaesthetics, and can be rapidly activated in response to forces sensed by the lipid bilayer of the plasma membrane, such as mechanical stretch and/or cell swelling [81,230,235,238,239,240]. Conversely, TREK-1 gating is inhibited by protein kinase A and protein kinase C phosphorylation pathways [235,236]. For a more in-depth review of K_2P_ channels, please refer to these recent review articles [231,232].

Mice with a genetic ablation of TREK-1 have heightened sensitivity to pain and heat, indicating that loss of TREK-1 enhances sensitivity to mechanical force [241,242,243], potentially due to TREK-1’s function in counteracting the activity of stretch-activated non-selective ion channels such as Piezo1 [232]. TREK-1 is inherently mechanosensitive, as patch clamp recordings of TREK-1 channels reconstituted in liposomes verified that it can activate in response to intrinsic tension of the membrane, in the absence of other proteins and cytoplasmic tethers [80,81] (Table 1). Changes to the lipid composition of the bilayer, which can alter fluidity and potentially curvature, can influence the activation energy threshold required to induce opening of the TREK-1 channel [233]. Arachidonic acid in particular has been identified as a lipid capable of modifying TREK-1 ion channel activity via modulation of membrane properties [230,244,245]. It has been proposed that within the lipid membrane, increased polyunsaturated fatty acids, such as arachidonic acid, may lower the lipid deformability barrier and promote an open state of this stretch-sensitive channel [233].

### 4.1. TREK-1 and Cardiac Remodelling

While TREK-1 functionality has predominantly been studied in the nervous system, this potassium channel has also been shown to be expressed and functional within the myocytes of rodents, pigs and humans [234,246,247], and fibroblasts of mouse hearts [234]. TREK-1 signalling plays an important role in normal sinoatrial node cell excitability [248]. It is also well established that cardiac TREK-1 dysregulation promotes development of AF, ventricular arrhythmias and HF [232,234,249,250,251,252].

In AF and HF patients, TREK-1 mRNA expression is strongly reduced in the atria [251]. Recapitulation of TREK-1 depletion, in a porcine model, further demonstrated a correlation between propensity towards AF and HF with TREK-1 depletion [251]. However, it should be noted that during a large scale analysis of TREK-1 expression in AF patients, Schmidt et al. only observed a non-significant trend in downregulation of TREK-1 expression [253].

Abraham et al. identified TREK-1 as a cardiomyopathy-related gene whose expression was increased in mouse hearts, including fibroblasts, following LV PO [234]. TREK-1 expression was also significantly increased during hypertrophy in the LV endocardium of rats following isoproterenol treatment [254]. However, isoproterenol is a known inhibitor of TREK-1, thus it is possible that the increase in TREK-1 expression was a compensatory mechanism due to chemical inhibition [254].

TREK-1 function within the heart has also been shown to protect against experimental MI-induced injury [252]. Permanent coronary artery ligation in mice lacking global TREK-1 expression developed larger-sized infarcts, greater LV diameter, and thinner posterior walls, indicating that TREK-1 function may protect against cardiac dysfunction during MI [252].

In summary, cardiac TREK-1 appears to play a homeostatic role in terms of electrical signalling, but may also influence pathological cardiac remodelling.

### 4.2. TREK-1 in Cardiac Fibroblasts

Through in vivo transgenic mouse studies, utilising global and inducible cell type-specific deletion of TREK-1, Abraham and colleagues were able to demonstrate that TREK-1 is an important modulator of cardiac hypertrophy, diastolic function and fibrosis in mouse hearts; with differential influence on cardiac dysfunction when TREK-1 is expressed in myocytes compared to fibroblasts [234]. Mouse models with global or CF-specific (*Tcf21* promoter-driven) TREK-1 deletion had greatly reduced cardiac fibrosis following TAC, although there was no apparent effect on CF-to-MF differentiation [234]. Preservation of cardiac function and chamber size with reduced interstitial fibrosis observed in the global TREK-1 KO was phenocopied in the CF-specific TREK-1 KO mice, but not the WT or myocyte-specific TREK-1 KO mice, indicating that the cardioprotective effect of global TREK-1 deletion was due to loss of TREK-1 expression specifically in fibroblasts [234]. Interestingly, stretch-induced, TGF-β- or EGF-treated ex vivo CF isolated from mice with global TREK-1 KO, had a significant reduction in JNK and c-Jun phosphorylation when compared to CF isolated from WT mice, whereas ERK1/2 and p38 signalling was unaffected [234]. This implicates TREK-1-mediated JNK signalling as being important in the hypertrophic and fibrotic response to PO.

In summary, although there is very little known about TREK-1 in CF, it appears that this potassium channel is an important regulator of CF function that underlies its role in cardiac remodelling (Figure 6C).

## 5. ATP-Sensitive Potassium Channels (K_ATP_)

K_ATP_ channels (Figure 6B) are hetero-octameric transmembrane channels that are inhibited by the intracellular nucleotides ATP and ADP [255]. It has been hypothesised that activation of K_ATP_ channels acts to reduce contractility of excitable cells during ATP depletion, as a means of preserving ATP bioavailability [256]. In addition to nucleotide depletion, K_ATP_ channels also activate in response to disruption of the F-actin cortical network [257,258] and mechanical stretch of the membrane [82].

K_ATP_ channels are formed by four pore-forming subunits: Kir6.1, Kir6.2, SUR1 and SUR2. RNA splicing of SUR2 subunits also give rise to further variants—SUR2A and SUR2B. Kir6.1 and Kir6.2 form the membrane-spanning regions responsible for the K^+^ inwardly rectifying channel and SUR1, SUR2A, or SUR2B subunits form the regulatory sulfonylurea receptor [255]. The assembly of subunits which form the K^+^ channel differs between cell types, and may confer different functional and pharmacological properties depending on which subunits are present [255]. Due to differences in K_ATP_ subunit expression and assembly between species, the efficacy of K_ATP_ agonists may also differ between species [259]. For this reason, a number of different agonists and antagonists, which each target different K_ATP_ subunits, have been utilised to study differential K_ATP_ channel assembly and function. For further general information on K_ATP_ channels please refer to the following review article [255].

Van Wagoner and colleagues first identified K_ATP_ as mechano-gated ion channels through negative pressure applied cell-attached and inside-out excised-patch recordings in neonatal and adult rat atrial myocytes. Single-channel K_ATP_ currents were detected as having a conductance of 52 pS in symmetric potassium solutions [82] (Table 1). They further demonstrated the mechanosensitivity of K_ATP_ channels through perforated patch whole-cell recordings of atrial myocytes, during hypotonic osmotic swelling of cells [82]. Negative pressure applied patch activation of K_ATP_ is dependent on the presence of the SUR subunit, suggesting this subunit is necessary for channel mechanosensitivity [260].

### 5.1. K_ATP_ and Cardiac Remodelling

There are three known subtypes of K_ATP_ channels found within the heart: cardiac mitochondria K_ATP_ (mK_ATP_), cardiac sarcolemma K_ATP_ (sK_ATP_), and plasma membrane K_ATP_ (pK_ATP_), with mK_ATP_ being 2000-fold more sensitive to the K_ATP_ agonist diazoxide than the other two [261,262]. Within mouse and rat hearts, K_ATP_ channels have been shown to be expressed in CM, CF, and in the SMC and EC of coronary arteries [262,263]. In rodents, K_ATP_ channels are expressed in CF isolated from normal heart tissue [264], but may not be functional under physiological conditions [265]. Within rats, the function of fibroblast K_ATP_ channels contribute towards cardiac remodelling and electrophysiology within the scar border zone following MI [265]. Using whole-cell patch clamp analysis of K_ATP_ current density, Benamer and colleagues were unable to detect K_ATP_ currents in CF isolated from normal rat heart tissue and tissue sampled from regions remote from the infarct zone [265]. However, K_ATP_ currents were detectable in CF isolated from the scar and border zone of an infarct scar [265], indicating that the K_ATP_ channel may only become active under pathological conditions.

In whole-heart analysis of both dogs and guinea pigs, the ability for K_ATP_ to shorten action potentials and reduce refractory periods was identified as having a pro-arrhythmic effects and promoted ischemic ventricular arrhythmias [266,267,268]. Despite the pro-arrhythmic effect of the channel, K_ATP_ functionality within the heart of guinea pigs has been suggested to protect against tissue damage as a result of reduced blood flow during ischemia/reperfusion injury [269]. Interestingly, ventricular CM isolated from Kir6.2 KO mice lose patch clamp-detected K_ATP_ currents, suggesting the Kir subunit is responsible for channel mechanosensitivity. Importantly, hearts from Kir6.2 KO mice were susceptible to more severe PO following TAC, with exaggerated fibrotic myocardial hypertrophy [270].

In summary, cardiac K_ATP_ channels play an important role in the heart. While functionality of K_ATP_ may provide cardioprotection against tissue damage caused by ischemia/reperfusion injury, it also appears to contribute to a pro-arrhythmic mechanism.

### 5.2. K_ATP_ in Cardiac Fibroblasts

K_ATP_ expression is increased in rat CF as they transdifferentiate into the MF phenotype, with K_ATP_ expression correlating with an increase in αSMA expression [265]. The increase in CF K_ATP_ expression, and functionality within the border region of an infarct scar, was postulated to contribute towards CF electrophysiological signalling in rat hearts; which occurred in response to reinfarction (the reoccurrence of symptoms of ischemia in patients or animals with a previous diagnosis of MI), and may have decreased the depolarising effect enacted on CM by CF [265].

Despite this descriptive evidence that K_ATP_ channel activity and expression correlate with development of the MF phenotype, numerous studies in both rodent and human CF, which utilise pharmacological modulators of K_ATP_ channels, indicate that CF K_ATP_ activity more likely opposes transdifferentiation of CF to MF [262,271,272]. Within cultured rat CF, K_ATP_ expression has been associated with MF maturation. Treatment of CF with the K_ATP_ activators diazoxide and pinacidil delayed MF maturation, while the K_ATP_ inhibitor glibenclamide increased MF maturation [262]. Schultz and co-workers found treatment of cultured human foetal CF with ursodeoxycholic acid hyperpolarised cells, downregulated αSMA expression, and prevented CF-to-MF differentiation [272]. This effect was attributed to an increase in K_ATP_ activity, as the hyperpolarising effect of ursodeoxycholic acid treatment was inhibited by glibenclamide [272]. In cultured rat CF, the K_ATP_ activator nicorandil attenuated Ang II-induced proliferation and endothelin-1 expression, an effect that was reversed by treatment of CF with glibenclamide, further indicating that K_ATP_ channels act to oppose the MF phenotype [271]. Rat CF mK_ATP,_ but not pK_ATP_, have also been reported to prevent transdifferentiation of CF to MF in ischemia pre-conditioned CF, following simulated ischemia-reperfusion injury [262]. Ischemic pre-conditioning reduced the rate of CF-to-MF differentiation in rats; a protective mechanism that was recapitulated by treatment of cells with the mK_ATP_ activator diazoxide, but not pinacidil, and was abolished by treatment of CF with the K_ATP_ inhibitor glibenclamide [262].

In summary, while CF K_ATP_ channels may act in reducing MF maturation and oppose pathological cardiac remodelling (Figure 6C), the mechanisms by which fibroblast K_ATP_ channels contribute to cardiac pathology require further investigation, and may be complicated by the intricacies of K_ATP_ subunit formation and distribution within the cell. Furthermore, it is currently unknown whether the mechanosensitivity of K_ATP_ channels contribute towards their function in CF biology. Thus, more nuanced genetic deletion models are required to fully understand the role of fibroblast K_ATP_ function in the heart.

## 6. Summary and Future Perspective

The complex regulation of mechanical ion channel signalling at the level of the CF is gradually being unravelled, with at least seven distinct mechanically activated cation channels identified that can modulate CF function at the levels of cell proliferation, MF differentiation, ECM turnover and paracrine signalling. While all these channels are activated in response to mechanical stimuli, only a small number (i.e., Piezo1 and TREK-1) are thought to be primary mechanosensors that detect mechanical stimulation in the absence of other cofactors. It is also important to consider that the net effect of mechanical stimulation on the CF cell will involve integrated (and sometimes opposing) signalling, not only from these ion channels, but also other cellular components including focal adhesion complexes and integrins, as well as surrounding ECM proteins. Moreover, the nature of the mechanical stimulus at the cellular level (e.g., stretch, compression, stiffness) adds a further layer of complexity. Thus, predicting the outcomes of CF mechanical signalling on cardiac physiology and various forms of pathological remodelling is far from straightforward. It must also be kept in mind that most cell culture studies are performed under conditions of high mechanical stiffness, due to the inherent rigidity of cell culture plasticware. This can have profound effects on CF function and may confound our ability to translate in vitro findings to the in vivo setting. Hence, to truly evaluate the physiological and pathophysiological roles of mechanically activated cation channels in CF in situ, it will be important to establish fibroblast-specific KO mice in which such channels are ablated, and then to assess the impact on normal cardiac physiology, as well as pathological cardiac remodelling. In recent years, several Cre-lox mouse models have been developed to enable fibroblast-targeted (e.g., Col1a2-Cre [273], Tcf21-Cre [274]) and MF-targeted (e.g., Postn-MCM [275]) KO of a variety of genes. Successful application of these approaches to study mechanosensitive ion channels in CF in vivo will help the field advance further and may reveal important therapeutic targets for reducing adverse cardiac remodelling. In doing so, we hope to be able to “channel the force to reprogram the matrix” and develop novel therapies for patients with fibrotic heart disease.

## Figures and Tables

**Table 1:** Summary of electrophysiological detection of ion channel mechanosensitivity.

Measurements of Mechanosensitivity in Cation Channels				
Channel	Model Used for Electrophysiological Reading	Nature of the Intervention	Channel Specific Signal Confirmed	Type of Mechanical Stimuli
TRPC6	Cell-attached patch [65,72]	Pipette applied negative pressure	No [65] Yes [72]	25% tonic mechanical stretch; membrane stretch Osmotic swelling; membrane stretch
	Excised patch readings of protein reconstituted in liposome [65,73]	Pipette applied negative pressure	Yes (spontaneously active but not stretch responsive)	
	Whole-cell patch [74]	Hypo-osmotic treatment	Yes, confirmed with inhibitor: SKF-96365	
TRPM7	Excised inside-out patch [75]	Pipette applied negative pressure; suction; osmotic swelling; perfusion-induced mechanical stress	Yes, confirmed in TRPM7 null cells; inhibitor: 2-APB; functional KO	Osmotic swelling; membrane stretch
	Whole-cell patch [76]			
TRPV1	Cell-attached patch [65]	Pipette applied negative pressure (not responsive)	No response detected	Osmotic swelling; membrane stretch
TRPV4	Cell-attached patch [65]	Pipette applied negative pressure (not responsive)	No response detected	Osmotic swelling; membrane stretch
	Whole cell [77]	Cell indentation with glass rod (no loss of reading with Trpv4 KO)	Yes, confirmed in cells isolated from Trpv4 KO mice +/−	
	Outside-out patch [77]	High-speed pressure clamp (no loss of reading with Trpv4 KO)	Yes, confirmed in cells isolated from Trpv4 KO mice +/− TRPV4 agonist	
Piezo1	Whole cell [78]	Cell indentation with glass rod	Yes, confirmed with siRNA knockdown	Membrane stretch
	Outside-out patch [79]	High-speed pressure clamp applied positive pressure	Yes, confirmed with siRNA knockdown	
TREK-1	Inside-out patch; outside out; cell attached [80]	Pipette applied negative pressure (No effect)	Yes (TREK-1 over expressed in COS cells)	Membrane stretch; cell swelling
	Inside-out patch of protein reconstituted in liposomes (channel spontaneously active) [81]	Pipette applied positive pressure (inactivates)	Yes, confirmed with TREK-1 inserted in liposome	
K_ATP_	Cell-attached; inside-out; excised-patch; perforated patch whole cell [82]	Negative pressure applied; hypotonic osmotic swelling	Yes, mechanosensitivity dependent on SUR subunit	Membrane stretch; cell swelling

## Data Availability

Not applicable.
